# Effects of Parents’ Smartphone Use on Children’s Emotions, Behavior, and Subjective Well-Being

**DOI:** 10.3390/ejihpe15010008

**Published:** 2025-01-13

**Authors:** Matea Bodrožić Selak, Marina Merkaš, Ana Žulec Ivanković

**Affiliations:** University Department of Psychology, Catholic University of Croatia, Ilica 244, 10000 Zagreb, Croatia; marina.merkas@unicath.hr (M.M.); azulec@unicath.hr (A.Ž.I.)

**Keywords:** technoference, smartphone use, parents, children, well-being

## Abstract

This study aimed to examine the associations between parents’ smartphone use during conversations with children, children’s emotional and behavioral reactions to parents’ smartphone use, and children’s well-being. This study was conducted on a sample of 284 children (aged 10 to 15 years, with a mean age of 12.23 in 2021; 40.2% boys). The data come from a four-wave longitudinal study (2021–2023) within the project D.E.C.I.D.E. Children reported how often their parents use smartphones during conversations with them (second wave), their emotions and behaviors related to parents’ smartphone use (third wave), and their subjective well-being (fourth wave). A proposed model was tested in which the frequency of parents’ smartphone use during parent–child conversations was a predictor, different children’s emotional and behavioral reactions to parents’ smartphone use were mediators, and children’s well-being was the criterion. The results showed that more frequent parents’ smartphone use is associated with more frequent children’s experiences of anger and sadness in situations when parents use smartphones while with children, which is linked to lower children’s well-being. More frequent parents’ smartphone use is associated with more giving up on seeking parents’ attention among children, which is related to lower well-being.

## 1. Introduction

The use of digital technology is becoming a new context for the growth and development of children and youth (Devine & Smith, 2023). According to Bronfenbrenner’s ecological theory (Bronfenbrenner, 1977), a child’s development is immersed in numerous systems and is interpreted in the context of multiple systems and their interactions. The first system, closest to the child, is the microsystem, which encompasses personal interactions between children and others in their immediate setting, typically consisting of family members (Bronfenbrenner, 1977). Navarro and Tudge (2023) refined Bronfenbrenner’s microsystem (Bronfenbrenner, 1977) by adapting it to digital technology. Namely, they assume that within the microsystem, there are two subsystems—virtual and physical (Navarro & Tudge, 2023). The virtual microsystem encompasses activities, social roles, and interactions that individuals experience in the virtual world using digital technology. In contrast, the physical microsystem refers to activities, social roles, and interpersonal interactions individuals encounter in face-to-face contexts (Navarro & Tudge, 2023).

When studying parent–child relationships in the context of technology use, two aspects need to be discussed. Firstly, parents serve as role models for technology use. When parents exhibit balanced and mindful tech habits, children are more likely to adopt similar behaviors (Nikken & Schols, 2015). Additionally, parenting styles and mediation can model children’s use and influence well-being. For example, parents who engage in discussions about technology use (e.g., online safety, appropriate content) foster better outcomes for children, including enhanced critical thinking and responsible digital behavior (Livingstone & Helsper, 2008). Setting strict rules about technology use may reduce risks (e.g., exposure to harmful content) but could also lead to conflicts between parents and children or covert use by children (Chen & Shi, 2019; Meeus et al., 2019). Secondly, technology use can either enhance or hinder parent–child communication. For example, video calls can help family members stay connected across distances, while excessive smartphone use by parents and children has been linked to technoference or disruptions in face-to-face interactions (McDaniel & Radesky, 2018a). Consequently, parents’ and children’s heavy use of social media and devices may reduce the quality and quantity of family interactions.

Parental attitudes toward digital technology use are also an important factor that is closely related to children’s behaviors, indicating that parental involvement might be crucial in managing screen time (Chia et al., 2022). Research shows that enhanced parent–child interactions can buffer the adverse effects of excessive digital device use on children’s executive function skills (Sadeghi et al., 2019). Sadeghi et al. (2019) found that parent–child interaction training not only improved the quality of relationships but also reduced children’s screen time.

While recent research often focuses on studying how children use smartphones and the effects of children’s smartphone use on their development (Griffith et al., 2024; Merkaš et al., 2024; Yoo, 2024), there is a smaller body of research on the effects of parental problematic smartphone use on child well-being (Hood et al., 2024; Mun, 2024; Tang et al., 2024). Parents use smartphones for various reasons, including (a) maintaining contact with others, fulfilling work obligations, and seeking information; (b) entertainment and distraction from stressful events; (c) as a coping strategy for unpleasant emotions, seeking support; (d) escaping stressful parenting situations (e.g., when a child has tantrums); and (e) for habitual checking of notifications (McDaniel, 2020). Regardless of the reason for smartphone use, parents’ smartphone use in the family context can result in technology interference during interactions with family members (e.g., children) (McDaniel, 2020; McDaniel & Coyne, 2016). Parents’ smartphone use and, subsequently, interference, can be understood as one of the marks of the child’s microsystem (Devine & Smith, 2023) influencing proximal processes and, consequently, the child’s development.

Technology interference (i.e., technoference) is a relatively recent concept referring to interruptions in face-to-face interactions due to smartphone use and is often documented in studies using various research methodologies (Kelly & Ocular, 2021; Lemish et al., 2020; McDaniel, 2015; McDaniel & Radesky, 2018a, 2018b; McDaniel et al., 2024a, 2024b). Technoference in close relationships refers to interruptions caused by technology that can occur through various digital devices (McDaniel, 2015; McDaniel et al., 2024a). Since smartphones are the most accessible and used devices (Ofcom, 2022), this paper focuses on technoference caused by smartphones. However, some studies have documented interruptions in communication caused by other devices such as tablets and TVs (McDaniel, 2015). Interruptions are often short, involving unintentional attention shifts from a person in an interaction toward the smartphone to check notifications (Frackowiak et al., 2023). Empirical studies showed that parents who engage in digital device use (e.g., smartphone use) while with children communicate less frequently with their children, are less responsive and sensitive to children’s needs and attention-seeking behaviors, feel less connected with their children, and may even become hostile after the child repeatedly tries to get their attention (Elias et al., 2021; Kildare & Middlemiss, 2017; McDaniel & Radesky, 2018b; Radesky et al., 2014, 2015). Studies further showed that children notice and react to parents’ digital device use and related interruptions in parent–child interactions (Hiniker et al., 2015; Merkaš et al., 2021; Porter et al., 2024; Steiner-Adair & Barker, 2013). Porter et al.’s (2024) study makes a notable contribution to the comprehension of how toddlers immediately react physiologically to technoference due to parents’ smartphone use, showing fluctuations in heart rate and respiratory sinus arrhythmia that mirror shifts in an emotional state and stress regulation. Qualitative studies show that children experience feelings of frustration, anger, and sadness in situations where parents are interrupted by technology (Merkaš et al., 2021; Steiner-Adair & Barker, 2013). Steiner-Adair and Barker (2013) found that children usually expressed unpleasant emotions when discussing the time spent with parents who used devices.

Numerous recent studies indicate the presence of negative effects of parents’ smartphone use on the quality of their interactions with children, as well as on children’s behavior and well-being (Braune-Krickau et al., 2021; Kılıman et al., 2024; Knitter & Zemp, 2020; Kushlev & Dunn, 2019; McDaniel, 2019; Merkaš et al., 2021; Tammisalo & Rotkirch, 2022). According to Kostić’s (2022) proposed model of technoference, technology interference has the potential to trigger conflicts in face-to-face interactions in which one person is engaged, as the individual not using the phone may perceive a sense of neglect or threat when the attention of the other person in the interaction is directed towards the mobile device. Such situations, which may or may not result in conflict, are associated with a decrease in relational well-being, as uncomfortable emotions and irritability from one partner can provoke similar responses in the other, especially feelings of being misunderstood during the interaction. Reduced relational well-being is linked to diminished personal well-being, which includes satisfaction across diverse life domains such as relationships, friendships, and family connections (Kostić, 2022).

Theoretically, the association between frequent exposure to technoference due to parents’ smartphone use and outcomes in younger children is usually explained in the context of attachment theory (Bowlby, 1969). On the other hand, emotional and behavioral responses in situations of technoference and their relations with developmental outcomes among older children and early adolescents can be interpreted from various theoretical perspectives. For example, the framework of the Sociological Theory of Symbolic Interactionism (Denzin, 2004) explains technology interference as a symbol of disconnection and disinterest. In the theory of Expectancy Violation Communication Theory (Burgoon, 1978), technoference is interpreted as a situation in which a person engaging in an interaction and using a smartphone fails to meet and/or violates expectations of behavior in interactions, leading to negative reactions. As a result, children resort to various behaviors aimed at regaining parental attention and establishing contact (Merkaš et al., 2021; Radesky et al., 2014, 2015). In other words, children start competing with smartphones for their parents’ attention (Radesky et al., 2014, 2015; Merkaš et al., 2021).

Researchers emphasize the importance of parental emotional availability during shared activities, as it facilitates the creation of secure attachment bonds, which in turn are associated with a range of positive outcomes in children and the quality of the parent–child relationship (Frackowiak et al., 2023; Lemish et al., 2020). Meeus and colleagues (2021) in their research found that children who perceive more technoference in their relationship with parents due to parental smartphone use rate the relationship as worse and report lower emotional support from parents. Nevertheless, if such patterns of behavior occur frequently, research suggests that children from these families are more susceptible to developing emotional and behavioral problems (Stockdale et al., 2018).

There is a lack of studies examining the behaviors and emotions of older children, namely, adolescents, in the context of parents’ smartphone use and related technoference. Most research in the field of technoference has relied on parental self-reports and mostly on parents of young children. However, aside from some studies suggesting that smartphone use is often automatic, meaning that this behavior is not always under our conscious control (Oulasvirta et al., 2012), research also indicates that parents’ and adolescents’ perspectives on technoference slightly differ (Davis et al., 2019). Davis et al. (2019) demonstrated a classic example of the fundamental attribution error (Fiske & Taylor, 1991) by showing that parents and adolescents legitimized their smartphone use while viewing the other’s smartphone use as the primary cause of interpersonal disruption. These perceptions may contribute to technology-related tensions in the family and conflict between parents and adolescents (Davis et al., 2019; Meeus et al., 2021). Since the perception of the person in the dyad who is not using the smartphone may be important in experiencing technoference, namely, their feelings during the interference, the current study uses children’s perceptions and evaluations of parental smartphone use during parent–child interactions. Furthermore, this research will address the gap in the literature wherein longitudinal studies linking at least two contexts of child development with some outcome variables related to overall child functioning are lacking (Devine & Smith, 2023).

**Figure 1:**
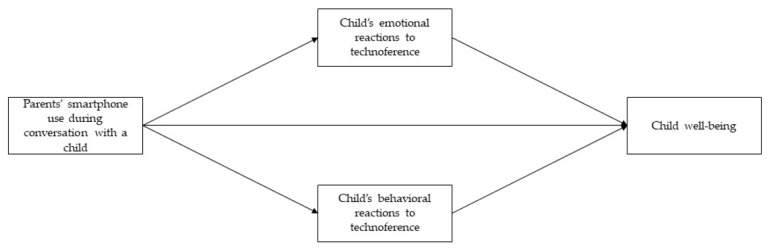
Proposed model of the effects of parents’ smartphone use through children’s emotional and behavioral reactions in situations of technoference on children’s well-being.

This study aimed to examine the associations between parents’ smartphone use during conversations with children, children’s emotional and behavioral reactions to technoference due to parents’ smartphone use, and children’s overall well-being. The proposed model is depicted in Figure 1. The proposed model was tested where the frequency of parents’ smartphone use during the parent–child conversation (second wave) was a predictor, different children’s emotional and behavioral reactions to technology interference due to parents’ smartphone use were mediators (third wave), and children’s well-being was the criterion (fourth wave).

**Hypothesis** **1.** *A higher frequency of parents’ smartphone use during parent–child conversation will directly and negatively contribute to children’s well-being*.

**Hypothesis** **2.** *A higher frequency of parents’ smartphone use during parent–child conversation will indirectly and negatively contribute to children’s well-being via an association with different children’s emotional reactions to technology interference due to parents’ smartphone use*.

**Hypothesis** **3.** *A higher frequency of parents’ smartphone use during parent–child conversation will indirectly and negatively contribute to children’s well-being via an association with different children’s behavioral reactions to technology interference due to parents’ smartphone use*.

## 2. Materials and Methods

### 2.1. Sample

The sample at wave 1 included 284 Croatian children (59.4% girls, 40.6% boys) who participated in the longitudinal study (S2) of the research project “Digital technology in the family: patterns of behavior and effects on child development” (D.E.C.I.D.E.). Children were aged 11 to 15, with a mean age of 12.64 (SD = 1.21) during the second wave of data collection, with their ages at wave 4 ranging from 11.58 to 16.58 (SD = 1.21). In terms of education, most fathers (58.7%) and mothers (49.1%) had completed high school, while a notable portion of fathers (37.3%) and mothers (48.4%) had obtained higher degrees. A small percentage of fathers (4%) and mothers (2.5%) had only completed elementary school. Regarding employment status, most of both fathers (80.1%) and mothers (73.1%) were employed full-time, with a minority of fathers (5.1%) and mothers (14.2%) being unemployed. Some fathers (11%) and mothers (11.6%) worked part-time, while a small percentage of fathers (3.7%) and mothers (1.1%) were retired. Most families in the sample were two-parent households (85.6%). Most of the parents had two children (50.2%), while 13.4% had one child, and 36.5% had more than two children. In terms of income, the monthly income for most families (66.9%) was below EUR 730.00 per family member, while one-third of families (33.1%) had a monthly income above this threshold. The range of monthly household income per person varied from below EUR 199.08 (1.2%) to above EUR 1260.87 (10.8%). In summary, most families included in this study were from middle to upper socioeconomic backgrounds.

Of the initial sample, 85.6% of children participated in the third wave of data collection (time 2 to time 3), 87.7% of children participated in the fourth wave of data collection (time 3 to time 4), and 78.9% of children participated in the study from time 2 to time 4. The time difference between each of the waves was approximately 5 to 6 months. A logistic regression analysis was used to test whether sample attrition can be predicted by the measured sociodemographic characteristics of children and parents included in this study. The results indicated that the attrition can be predicted only based on the child’s age (χ2 (5) = 46.71, *p* < 0.01; b = 1.098, *p* < 0.01). Older children dropped out of this study, which was expected since our initial sample consisted of children attending the final grade of primary school, and it was more difficult to reach them and collect their data at later measurement points when they transitioned to high school or left the educational system.

### 2.2. Measures

Given the length of the questionnaire and the purpose and goals of the project from which data for this study were taken, not all variables were measured at all waves in the S2 study. In this paper, we use measures applied in waves 2, 3, and 4. Measures related to children’s perception of parents’ smartphone use and their behaviors and emotions in situations of technoference were measured only once during the S2 study. The term ’mobile phone’ was used in this study because, in the Croatian language, children use ’mobile phone’ and ’smartphone’ as synonyms. Therefore, mobile phone and smartphone are used interchangeably in this study and this manuscript.

1. Parents’ smartphone use during conversations with children was assessed in the second wave of the S2 study using a newly constructed scale for the project’s purposes. The scale consists of 5 items (e.g., “During conversations with you, your parents (1) text on their phones, (2) answer incoming calls, (3) hold their phones in their hands, (4) glance at their phones, (5) check notifications on their phones.”). Children were asked to rate how often their parents use a mobile phone during conversations with them, using a scale from 1 (never) to 5 (always), with 2 (rarely), 3 (sometimes), and 4 (often) as intermediate options. Exploratory factor analysis (EFA) revealed a one-factor structure of the scale, explaining 61.22% of the construct’s variance. The Cronbach’s alpha for the entire scale is α = 0.88. Items were averaged to obtain a total score, with higher scores indicating more frequent use of smartphones by parents during conversations with their children.

2. Children’s emotional reactions in situations of technoference due to parents’ mobile phone use were measured using a scale constructed for the project’s purposes in the third wave of the longitudinal study. Items were developed and constructed based on the children’s answers in focus groups and interviews in Study 1 of the same project. The scale consists of 13 items (“When you talk to your parents about something important and they use their mobile phone, and you feel like they didn’t hear you. How often do you (1) get upset, (2) feel indifferent, (3) get angry, (4) feel ignored, (5) be okay with it, (6) be indifferent, (7) get annoyed, (8) get sad, (9) not mind it, (10) want it to stop immediately, (11) feel nothing, (12) feel insignificant, (13) not get too upset about it?”). Children were asked to rate how often their parents use a mobile phone during conversations with them, using a scale from 1 (never) to 5 (always), with 2 (rarely), 3 (sometimes), and 4 (often) as intermediate options. The results of the exploratory factor analysis (EFA) indicate a three-factor structure. The first factor pertains to items assessing the frequency of experiencing anger, explaining a total of 36.13% of the variance (α = 0.87). The second factor relates to items concerning indifference, explaining 17.71% of the variance (α = 0.86), and the third factor pertains to the emotion of sadness, explaining an additional 4.57% of the variance (α = 0.76). Items were averaged to obtain a total score for each component, with higher scores indicating more frequent experiencing of emotional responses in situations where technology interference due to parental mobile phone use occurs.

3. Children’s behavioral reactions in situations of technoference due to parents’ mobile phone use were assessed using a scale constructed for the project’s purposes in the third wave of the longitudinal study. Items were developed and constructed based on the children’s answers in focus groups and interviews in Study 1 of the same project. The scale consists of a total of 16 items, with responses ranging from 1 (never) to 5 (always), with the intermediate values being 2 (rarely), 3 (sometimes), and 4 (often) (“When you talk to your parents about something important to you and they use their mobile phone, and you feel like they didn’t hear you. How often do you (1) shout, (2) send a message to your parent on their mobile phone, (3) wait for them to finish, (4) go do something else, (5) raise your voice, (6) wait for them to pay attention to you, (7) continue with your activities, (8) touch your parent (e.g., touch their shoulder), (9) do nothing, (10) wait for them to address you, (11) talk about something trivial, (12) come closer your parent, (13) patiently wait for them to address you, (14) leave your parent without attempting to call them multiple times, (15) speak loudly, (16) wait because you know they will address you?”). The results of the EFA indicate a four-factor structure. The first factor relates to behaviors where children patiently wait for their parents’ response, explaining a total of 23.08% of the variance (α = 0.82). The second factor pertains to behaviors where children attempt to gain their parents’ attention through verbal efforts, explaining a total of 16.57% of the variance (α = 0.74). The third factor concerns behaviors involving physical contact with the parent to re-establish contact, explaining an additional 7.47% of the variance (α = 0.56). The fourth factor relates to behaviors indicating giving up on trying to gain the parent’s attention, explaining an additional 3.20% of the variance (α = 0.700). Items were averaged into a total score for each component, with higher scores indicating more frequent behavioral reactions in situations of technology interference due to parental mobile phone use.

4. Children’s negative and positive affect were measured using the shortened version of the PANAS Scale (Mackinnon et al., 1999) in the fourth wave of the longitudinal study. Participants rated the frequency of positive and negative affect over the past two weeks on a scale from 1 (not at all or very little), 2 (little), 3 (moderately), 4 (quite a bit), to 5 (very much). The factor analysis revealed a two-factor solution—positive affect (i.e., excited, enthusiastic, alert, inspired, determined) and negative affect (i.e., distressed, upset, scared, nervous, afraid). The items comprising the negative affect factor explained 30.91% of the variance, while items loaded on positive affect explained 19.01% of the variance. The Cronbach’s Alpha coefficient was α = 0.78 for the negative affect (5 items) and α = 0.84 for the positive affect (5 items) subscale. Average scores were computed separately for positive (PA) and negative (NA) affect, with higher scores indicating greater emotional intensity of PA and NA in adolescents.

5. Children’s life satisfaction was assessed using the Brief Multidimensional Students’ Life Satisfaction Scale (BMSLSS) (Huebner, 1994; Seligson et al., 2003) in the fourth wave of the longitudinal study. The scale comprises five items, each addressing different life domains (family, friends, school, self, living environment), as well as one item covering global life satisfaction. Participants indicated how satisfied they are with each domain and their overall life using a scale from 1 (completely unsatisfied), 2 (mostly unsatisfied), 3 (somewhat unsatisfied), 4 (neither unsatisfied nor satisfied), 5 (somewhat satisfied), 6 (mostly satisfied), to 7 (completely satisfied). Factor analysis revealed a one-factor solution with this factor explaining 45.58% of the variance. The Cronbach’s Alpha coefficient for the five items measuring satisfaction in five life domains was α = 0.79. Scores from these five items were averaged to derive an overall life satisfaction score, with higher scores indicative of greater life satisfaction among adolescents.

### 2.3. Procedure

The data used for this paper were drawn from the second, the third, and the fourth waves of the longitudinal study (Study 2) (wave 2: May–June 2022; wave 3: November–December 2022; wave 4: April–June 2023) of the research project “Digital technology in the family: patterns of behavior and effects on child development” (D.E.C.I.D.E.). Ethical clearance for this study was obtained from the Ethical Committee of the Catholic University of Croatia, and it was approved to be conducted in elementary schools by the Ministry of Science and Education of Croatia. Given that participation in this study was voluntary, the selection of schools and participation was convenient. A total of 22 schools located in the research area, the city of Zagreb and Zagreb County, Croatia, were contacted via email, 6 of which agreed to participate in this study via their principals signing a consent form. A total of 1096 children were approached through the participating schools, with informed consent forms and parental questionnaires distributed. The response rate was 26%. Following the signing of consent forms by participating parents (83% being mothers) and children (N = 284; comprising 59.4% girls and 40.6% boys) at their residences, data collection occurred partly in school settings during regular classes and partly through the completion of questionnaires by children and parents at home, given the extensive length of the questionnaires at each wave. The questionnaire was administered by school psychologists and the project team, with group administration sessions lasting approximately 20 to 30 min. Each child and parent who participated in this study received a gift voucher for the local zoo.

### 2.4. Data Analysis

Descriptive statistical parameters (means, standard deviations, minimum and maximum observed results) were calculated using the SPSS 26.0 software package (Table 1). Correlations between the study variables were computed using Pearson correlation coefficients (Table 2).

To address the set objective, two models were tested: one in which the mediating variable is children’s emotional reactions in technoference situations due to parents’ smartphone use, and the other in which the mediator is children’s behavioral reactions in technoference situations due to parents’ smartphone use. Given the relatively small sample size in this study, model simplicity and statistical robustness were prioritized. Two models were tested: one for emotional reactions and another for behavioral reactions.

**Figure 2:**
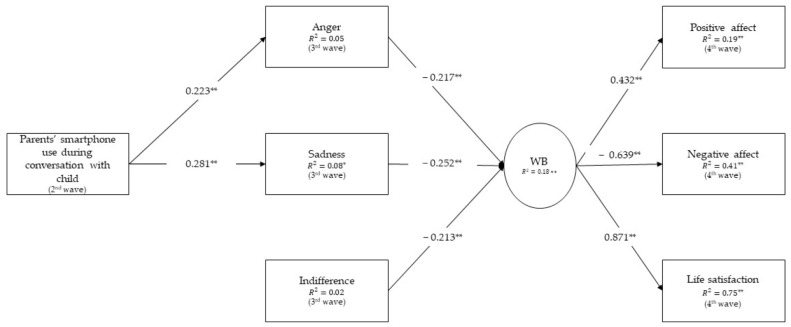
The effects of parents’ smartphone use through children’s emotional reactions in situations of technoference on children’s well-being: the results derived from testing the final model. Note: ** *p* < 0.01; * *p* < 0.05.

**Figure 3:**
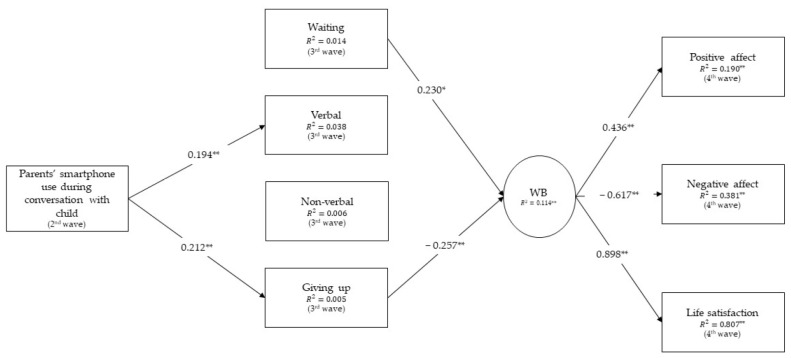
The effects of parents’ smartphone use through children’s behaviors in situations of technoference on children’s well-being: the results derived from testing the final model. Note: ** *p* < 0.01; * *p* < 0.05.

Structural equation modeling was employed to test the assumed models, with two models tested (one for children’s emotional reactions and the other for children’s behavioral reactions) using Mplus (Muthén et al., 2017). Specifically, the models examined the direct effects of parents’ smartphone use during conversations with their child on the child’s well-being, as well as the indirect effects of parents’ smartphone use during conversations with their child, mediated by the child’s emotional (Model 1, Figure 2) and behavioral (Model 2, Figure 3) reactions in situations of technoference due to parents’ smartphone use, on the child’s well-being. Model 1 (Figure 2) involved one direct effect of parents’ smartphone use during conversations with their child on the child’s well-being, as well as the indirect effects of parents’ smartphone use during conversations with their child, through the child’s emotional reactions in situations of technoference due to parents’ smartphone use, on the child’s well-being (three effects). Model 2 (Figure 3) involved one direct effect of parents’ smartphone use during conversations with their child on the child’s well-being, as well as the indirect effects of parents’ smartphone use during conversations with their child, through the child’s behavioral reactions in situations of technoference due to parents’ smartphone use, on the child’s well-being (four effects). The bootstrap method (N = 1000 samples) was used to estimate the magnitude of the effects and the associated confidence intervals in the final model.

Since no statistically significant correlations were found between most behavioral and emotional reactions and sociodemographic variables (namely, age and gender; Table 2), we did not include sociodemographic variables as covariates in the tested models. The inclusion of covariates would also complicate model testing due to the need for extensive parameter estimation.

## 3. Results

### 3.1. Descriptive Analysis and Correlations

Table 1 presents descriptive statistics for the variables used in this study. According to the descriptive statistics, it can be said that children rated their parents as rarely using smartphones during conversations with them, on average. Children reported experiencing anger, indifference, and sadness rarely, on average, when speaking to their parents about something important while the parent was on the phone. In these situations, children reported on average waiting for their parent to pay attention to them most often, while verbally calling out to them was the least frequently reported behavior. On average, children exhibited moderate positive affect and less pronounced negative affect and were generally satisfied with their lives.

Table 2 shows the Pearson correlation coefficients between the study variables. Parents’ smartphone use during conversations with their children is positively associated with feelings of anger and sadness, as well as with behaviors of verbal attempts to gain parental attention and behaviors of giving up in situations where the child talks to the parent about something important while the parent is using a smartphone. Feelings of anger and sadness in situations of technology interference due to parents’ smartphone use, as well as behaviors of verbal attempts to establish contact with parents and behaviors of giving up on establishing contact are positively correlated with negative affect and negatively correlated with life satisfaction.

### 3.2. Model Testing

We first tested the proposed model of the direct and indirect effects of parents’ smartphone use during conversations with children through children’s emotional reactions in situations of technoference due to parents’ mobile phone use on children’s well-being (Figure 2). The model showed good fitting to the data (n = 200; X^2^ = 13.86, df = 9, *p* = 0.127, CFI = 0.981, TLI = 0.955, SRMR = 0.043, RMSEA = 0.052, C.I. 95% [0.000–0.103]). The results showed that there are no significant paths from parents’ smartphone use during a conversation with the child (a) to the emotion of indifference in the situation of technoference due to parents’ smartphone use (β = −0.041, *p* = 0.578), and (b) to the child’s well-being (β = −0.033, *p* = 0.687). Thus, these paths were removed from the model, and the trimmed model was tested again.

The final trimmed model is shown in Figure 2. This model (Figure 2) showed a good fit to the data (n = 200; X^2^ = 14.169, df = 10, *p* = 0.165, CFI = 0.983, TLI = 0.967, SRMR = 0.044, RMSEA = 0.046, C.I. 95% [0.000–0.196]). The indirect effects of parents’ smartphone use during conversations on the child’s well-being via anger (β = −0.021, *p* = 0.000; 95% CI [−0.052, 0.000]) and sadness (β = −0.027, *p* = 0.000; 95% CI [−0.067, −0.001]) were found to be significant.

Next, we tested the proposed model of the direct and indirect effects of parents’ smartphone use during conversations with children through children’s behavioral reactions in situations of technoference due to parents’ mobile phone use on children’s well-being (Figure 3). The model showed good fitting to the data (n = 200; X^2^ = 14.759, df = 10, *p* = 0.141, CFI = 0.975, TLI = 0.931, SRMR = 0.042, RMSEA = 0.049, C.I. 95% [0.000–0.98]). The results showed that there are no significant paths from parents’ smartphone use during conversations with the child (a) to the behavior of waiting in the situation of technoference (β = −0.117, *p* = 0.101), and (b) to non-verbal behaviors in the situation of technoference (β = 0.080, *p* = 0.266). The results showed that there are no significant paths from (a) verbal behaviors (β = −0.116, *p* = 0.221), and (b) non-verbal behaviors (β = −0.014, *p* = 0.876) in the situation of technoference to children’s well-being. There was no significant direct path from parents’ smartphone use during conversations with their child to the child’s well-being (β = −0.031, *p* = 0.705). All mentioned paths were removed from the model, and the trimmed model was tested again.

The final trimmed model is shown in Figure 3. This model (Figure 3) showed a good fit with the data (n = 200; X^2^ = 14.902, df = 11, *p* = 0.187, CFI = 0.980, TLI = 0.949, SRMR = 0.042, RMSEA = 0.042, C.I. 95% [0.000–0.091]). The indirect effect of parents’ smartphone use during conversations with their child on the child’s well-being via behaviors of giving up from trying to recall parents’ attention (β = −0.030, *p* = 0.000; 99% CI [−0.080, −0.002]) was found to be significant.

## 4. Discussion

The aim of this study was to examine the associations between parents’ smartphone use during conversations with children, children’s emotional and behavioral reactions to technoference due to parents’ smartphone use, and children’s subjective well-being. The results showed that parents’ smartphone use during conversations with children is associated with children’s experiences of anger and sadness in situations of technoference due to parents’ smartphone use, which is linked to poorer children’s well-being. Additionally, more frequent parents’ smartphone use is associated with a higher reported behavior of giving up on trying to recall parents’ attention in children, which is related to lower levels of children’s well-being. Thus, Hypothesis 1 “Higher frequency of parents’ smartphone use during parent–child conversations will directly and negatively contribute to children’s well-being” was not conformed. Hypothesis 2 “A higher frequency of parents’ smartphone use during parent–child conversations will indirectly and negatively contribute to children’s well-being via an association with different children’s emotional reactions to technology interference due to parents’ smartphone use” was partially confirmed. Hypothesis 3 “A higher frequency of parents’ smartphone use during parent–child conversations will indirectly and negatively contribute to children’s well-being via an association with different children’s behavioral reactions to technology interference due to parents’ smartphone use” was partially confirmed.

The results of this study align with findings from rare previous research indicating that children experience unpleasant emotions such as sadness and anger in situations of technoference (Merkaš et al., 2021; Steiner-Adair & Barker, 2013). In this study, parents’ smartphone use was associated with more frequent feelings of sadness and anger in situations of technoference, which was then linked to poorer child well-being. Additionally, the more children reported feeling indifferent in situations of technoference due to parents’ smartphone use, the poorer their well-being was. These results can be interpreted within the displacement hypothesis (Valkenburg & Peter, 2007), suggesting that parental attention during technoference shifts from the child to the device, leading children to feel ignored when they are expressing something important to their parents. In such scenarios, children may interpret their parents as inaccessible and less attentive, impeding the formation of healthy relationship bonds. Specifically, to maintain and foster quality bonds, parents need to be consciously engaged and attuned to the child’s (non)verbal cues, responding promptly (Ainsworth et al., 1974). However, with technoference, parental attention is divided between the smartphone and the child, overloading cognitive resources and obstructing timely and responsive interactions with the child’s bids (Linder et al., 2021; Zayia et al., 2021). Specifically, one of the consequences of technoference is the feeling of being ignored by the other person in the dyad because the person using the smartphone implicitly sends a message to the person with whom they are interacting in person that they are not important (McDaniel & Coyne, 2016). The results of this study support this proposition in parent–child relationships.

In situations of technoference, children resort to various behaviors to attract parents’ attention, often resorting to verbal attempts, such as raising their voice to recall their parents’ attention. The results indicate that children often give up trying to get their parents’ attention or may not attempt to do so at all. Indeed, giving up and not attempting to get their parents’ attention is associated with poorer child well-being later. In situations where children are trying to communicate something important to their parents, but the parents are not paying attention to them because they are immersed in using digital technology devices, they implicitly may convey to the children that they are not important at that moment. As a result, children may feel neglected and simply give up on trying to get their parents’ attention. Research conducted by Stockdale and colleagues (Stockdale et al., 2018) suggests that children in families where technoference frequently occurs are at a higher risk of developing psychological problems later in life. In that context, it is possible that children who give up may have learned from past experiences that their efforts to gain attention are unsuccessful, leading to behaviors of powerlessness and potentially impacting their overall well-being. Namely, according to learned helplessness theory (Nolen-Hoeksema et al., 1986), these children who do not even attempt to get their parents’ attention may feel as if they have no control over the situation they are in, resulting in motivational deficits (lowered response initiation and lowered persistence), cognitive deficits (inability to perceive existing opportunities to control outcomes), and, in humans, emotional deficits (sadness and lowered self-esteem) (Maier & Seligman, 1976; Nolen-Hoeksema et al., 1986).

Conversely, waiting behaviors in situations of technoference are associated with better child well-being later. It is possible that children who wait for a response from their parents in technoference situations generally perceive their relationship with their parents as more positive and the quality of their relationship as higher. The quality of the relationship in this context may be an important factor that needs to be considered when interpreting the emotional and behavioral reactions in situations of technoference as well as parent–child relationships in the context of technoference. Therefore, it is important to consider not only the immediate emotional and behavioral responses of children to technoference but also the broader context of their relationship with their parents.

In this study, life satisfaction emerged as the well-being indicator most strongly explained by the model. This may be attributed to the fact that affect, as a short-term variable and the emotional component of subjective well-being, is influenced by immediate daily factors for the children, such as school-related stress (e.g., receiving a low grade that day) or conflicts with friends. In contrast, life satisfaction is a more stable measure and represents the cognitive component of subjective well-being (Huebner, 2004). Crucial to the attainment of life satisfaction in adolescents are adaptive and supportive familial and environmental conditions, including family structure, parenting style, parental emotional and social support, family conflict, and the quality of the physical environment (Proctor et al., 2017). The findings of this study, therefore, support the claim that family circumstances—in this case, parents’ smartphone use—play a significant role in shaping children’s life satisfaction. The results indicate that, within the family context, children’s negative reactions to parents’ smartphone use significantly contribute to lower life satisfaction among children. This suggests that, over time, family interactions and relationships disrupted by technology use can have a negative impact on children’s life satisfaction.

### Limitations and Future Directions

This study presents certain limitations that warrant consideration. Firstly, the study sample consisted of voluntary participants, potentially resulting in sample bias and affecting the generalizability and stability of the findings. To address this, we recommend validating the findings across culturally and demographically diverse samples of children from various family backgrounds. It is also important to consider the context of the interaction in which technoference occurs. Namely, although research suggests that technoference due to parents’ smartphone use most commonly occurs during leisure activities or time spent with the child, providing parents with the opportunity to create a stimulating environment for the child to develop attachment and bonding (McDaniel, 2020), it is important to consider the characteristics of this context. Some studies indicate that simply stating the reasons for smartphone use may mitigate the negative effects of technoference (McDaniel & Wesselmann, 2021). Considering that, future studies should explore the effectiveness of different strategies, such as openly communicating the reasons for smartphone use or setting boundaries when it comes to device usage, in mitigating the negative effect of technology interference on parent–child attachment. This could involve conducting experimental studies where parents are randomly assigned to different intervention groups and their interactions with their children are observed and assessed over time.

Furthermore, factors such as parental attitudes towards technology, family dynamics, the quality of parent–child relationships, and child temperament may interact with parental smartphone use while with children. Longitudinal studies tracking parent–child interactions and attachment development could also provide valuable insights into the long-term effects of technoference on parent–child relationships. For future studies, it is suggested that the two tested models in this paper be combined into a single model for testing the effects, as proposed in the introduction. Since children’s emotional reactions are correlated with their behavioral reactions, it would be valuable to account for these associations within the same model. Furthermore, sociodemographic variables should be included as covariates in future models. It is possible that girls and boys differ in their reactions to the parental behavior of using technology. Perhaps the family’s socioeconomic status also affects the behavior of parents and children when using technology.

## 5. Conclusions

The results showed that more frequent parents’ smartphone use is associated with more frequent children’s experiences of anger and sadness in situations of face-to-face conversation, which are linked to poorer children’s well-being. Additionally, more frequent parents’ smartphone use is associated with a higher reported behavior of giving up on trying to recall parents’ attention among children, which is related to lower levels of well-being. The findings of this study provide a theoretical evidence-based foundation for designing preventive programs aimed at addressing problematic smartphone usage patterns in the family context, emphasizing the important role of parental behavior and children’s perceptions of that behavior in the overall well-being of children. Educating parents and children about healthy technology habits seems to be an important factor that could eliminate or mitigate the negative consequences of technology use on parent–child relationships and children’s well-being.

## Figures and Tables

**Table 1:** Descriptive statistics for the study variables.

	Study variable	N	M	SD	Min–Max	Med
	1. Parents’ smartphone use during a conversation with the child (2nd wave)	235	2.05	0.91	1–5	1.8
Child’s emotional reactions in situations of technoference (3rd wave)	2. Anger	223	2.42	1.05	1–5	2.25
	3. Indifference	220	2.41	0.91	1–5	2.33
	4. Sadness	225	2.24	0.96	1–5	2.33
Child’s behavioral reactions in situations of technoference (3rd wave)	5. Waiting	224	3.09	0.90	1–4.83	1.17
	6. Verbal behaviors	224	1.96	0.83	1–5	1.18
	7. Non-verbal behaviors	224	2.39	0.85	1–5	2.33
	8. Giving up	223	2.39	0.89	1–5	2.33
Child’s well-being (4th wave)	9. Positive affect	197	3.18	0.79	1–5	3.20
	10. Negative affect	199	2.13	0.88	1–5	2.00
	11. Life satisfaction	191	5.61	1.09	2.60–7	6.00

**Table 2:** Correlations among the study variables.

		1	2	3	4	5	6	7	8	9	10	11	12	13
	1. Parents’ smartphone use during a conversation with child (2nd wave)	-	0.220 **	−0.039	0.276 **	−0.113	0.184 *	0.078	0.194 **	0.071	0.139	−0.123	−0.010	0.100
Child’s emotional reactions in situations of technoference use (3rd wave)	2. Anger		-	−0.245 **	0.667 **	−0.230 **	0.615 **	0.295 **	0.175 **	0.019	0.247 **	−0.271 **	−0.019	−0.018
	3. Indifference			-	−0.329 **	0.494 **	−0.004	0.048	0.383 **	−0.010	0.075	−0.092	0.038	0.112
	4. Sadness				-	−0.173 **	0.428 **	0.307 **	0.135 *	−0.033	0.307 **	−0.270 **	−0.116	−0.133 *
Child’s behavioral reactions in situations of technoference (3rd wave)	5. Waiting					-	−0.202 **	0.200 **	0.341 **	0.059	−0.062	0.122	0.002	−0.059
	6. Verbal behaviors						-	0.275 **	0.223 **	0.037	0.191 **	−0.220 **	0.033	0.121
	7. Non-verbal behaviors							-	0.165 *	0.041	0.113	−0.014	0.030	−0.103
	8. Giving up								-	0.080	0.239 **	−0.169 *	−0.018	0.090
Child’s well-being (4th wave)	9. Positive affect									-	−0.216 **	0.403 **	0.232 **	−0.015
	10. Negative affect										-	−0.569 **	−0.107	−0.062
	11. Life satisfaction											-	0.127	−0.020
Sociodemographic variables	12. Gender												-	0.067
	13. Age													-

Note: * *p* < 0.05; ** *p* < 0.001; Gender 1—female, 2—male.

## Data Availability

The data that support the findings of this study are available from the corresponding author, Marina Merkaš, upon reasonable request.
